# Hematological and biochemical reference intervals for populations of seabird species from an Archipelago in Rio de Janeiro, Brazil

**DOI:** 10.3389/fvets.2026.1749869

**Published:** 2026-03-02

**Authors:** Joana Maçaira, Marthiellen Roosevelt de Lima Felix, Amanda de Oliveira Alcântara, Paula Maçaira, Larissa Schmauder Teixeira Da Cunha, Marina Galvão Bueno, Aline Moreira de Souza

**Affiliations:** 1 Laboratory of Compared and Environmental Virology, Oswaldo Cruz Foundation (Fiocruz), Rio de Janeiro, Brazil; 2 Department of Veterinary Clinic and Pathology, Universidade Federal Fluminense, Niterói, Brazil; 3 Department of Industrial Engineering, Pontificia Universidade Catolica do Rio de Janeiro, Rio de Janeiro, Brazil; 4 Instituto Mar Adentro, Rio de Janeiro, Brazil

**Keywords:** biochemistry, free-ranging, *Fregata magnificens*, hematology, *Sula leucogaster*, veterinary clinical pathology, wildlife

## Abstract

**Introduction:**

Establishing reliable hematological and biochemical reference intervals for seabirds is essential for health assessment, rehabilitation, and conservation programs. However, baseline physiological data for these animals remain scarce. This study aimed to determine hematological and biochemical reference intervals for two free-ranging seabird's species commonly found along the coast of Rio de Janeiro, Brazil: the brown booby, *Sula leucogaster* (Boddaert, 1783), and the magnificent frigatebird, *Fregata magnificens* (Linnaeus, 1758).

**Methods:**

Between March 2024 and May 2025, blood samples were collected from apparently healthy individuals in the Natural Monument of the Cagarras Islands Archipelago (MoNa Cagarras), the main breeding areas for these species in the State of Rio de Janeiro. Hematological and biochemical parameters were analyzed using standard laboratory procedures. Reference intervals were established following the guidelines of the American Society for Veterinary Clinical Pathology.

**Results:**

Overall, hematologic parameters for both species were similar to those previously reported for individuals maintained in rehabilitation centers. The most notable differences were observed in total thrombocytes and white blood cell counts, which presented higher values. Regarding biochemical parameters, uric acid, and creatine kinase levels were higher in brown boobies. Conversely, alanine aminotransferase, aspartate aminotransferase, and albumin levels were lower when compared to other reports. In frigatebirds, alanine aminotransferase levels were also elevated, but other parameters showed little variation.

**Discussion:**

For both brown boobies and magnificent frigatebirds, this study presented higher values in total thrombocytes and white blood cell counts, as well as uric acid and creatine kinase levels. These findings possibly reflect capture-related physiological responses from wild individuals. The reference intervals described here represent the first *in situ* values established for these two seabird species from the MoNa Cagarras in Brazil, providing essential baseline data for health and clinical evaluation, rehabilitation monitoring, and the conservation of tropical seabirds, while highlighting the importance of assessing the health of wild animals within their natural habitat.

## 1 Introduction

Seabirds can be considered effective bioindicators of marine ecosystem health due to their ecological characteristics, such as high trophic position and colonial nesting habits (1). Description of disease and zoonotic infections in these animals have increased, reinforcing the need for long-term health monitoring of populations, particularly those inhabiting coastal areas in proximity to human environments (2, 3). Normally, the health status of wildlife populations is based on parameters such as size, reproductive success of colonies and/or on annual survival rates (4). However, these indicators often reflect problems only after they have potentially compromised conservation efforts. Therefore, a more preventive strategy, such as establishing hematological and biochemical reference intervals (RIs) in these species, would provide a more accurate assessment of their health status (5). Furthermore, defining these intervals enables clinicians not only to detect early subclinical processes, but also to guide therapeutic decisions and pre-release management protocols (6). Unfortunately, studies establishing reference values for wildlife species, particularly under *in situ* conditions, are scarce and often challenging to conduct or standardize, especially when the analysis requires a large number of individuals to be sampled (7).

In this context, at least 100 seabird species have been reported along the Brazilian coastline, representing approximately 25% of all known seabird species worldwide (8). Populations of *Sula leucogaster* (Boddaert, 1783), brown booby, and *Fregata magnificens* (Linnaeus, 1758), magnificent frigatebird, are frequently found in mixed colonies and represent part of the order Suliformes in Brazil. These species nest and breed on oceanic islands along the Brazilian coast, roughly between 21°S and 29°S on the South and Southeast region, comprising approximately 16 nesting sites for brown boobies and 10 for magnificent frigatebirds (9). Particularly in the southeastern region of Brazil, these colonies occur on islands located near densely populated areas, where birds are exposed to anthropogenic stressors such as polluted wastewater discharge (10). Consequently, these seabirds are considered important environmental sentinels of their habitat.

However, to date, there is no study assessing the health status of these populations *in situ*, nor establishing RIs for hematological and biochemical parameters for these species in this region, which highlights the relevance of the present study.

In this study, we established hematological and biochemical RIs for populations of *S. leucogaster* and *F. magnificens* breeding in the Natural Monument of the Cagarras Islands Archipelago (MoNa Cagarras), a Federal Conservation Unit in Rio de Janeiro city and one of the largest nesting sites for magnificent frigatebirds in the South Atlantic. Additionally, we provide representative images of blood cell morphology from both species to support hematological characterization.

## 2 Methodology

### 2.1 Ethical approval

All animal capture, handling, release, and sample collection procedures complied with national and regional legislation and were authorized by the Animal Use Ethics Commission of the Oswaldo Cruz Foundation (CEUA L-019/2021-A3), the Chico Mendes Institute for Biodiversity Conservation - ICMBio (SISBio 73163-8) and the National System for the Management of Genetic Heritage and Associated Traditional Knowledge (SISGEN A918B14 and A5A95E3).

### 2.2 Animal capture and sample collection

**Figure 1:**
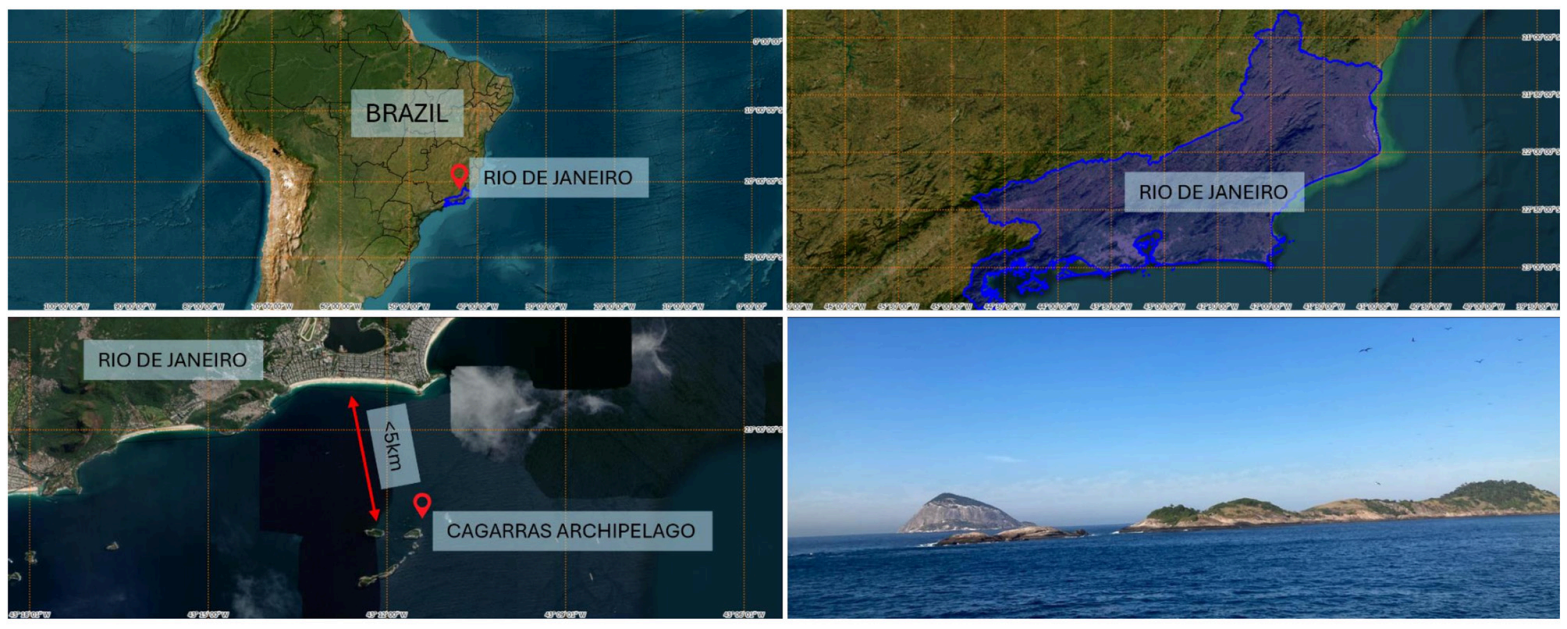
Geographical location of Cagarras Archipelago, Rio de Janeiro — RJ, Brazil, capture and sampling of brown boobies, *Sula leucogaster* (Boddaert, 1783) and magnificent frigatebirds, *Fregata magnificens* (Linnaeus, 1758) for establishing hematologic and biochemical reference intervals. Source: Brazilian Institute of Geography and Statistics (IBGE, 2024). Map base obtained from https://www.ibge.gov.br/apps.basescartograficas/.

Between March 2024 and May 2025, field activities were conducted monthly on Cagarra and Redonda Islands, part of the MoNa Cagarras in Rio de Janeiro, Brazil (23°01′49^′′^S 43°12′01^′′^W) (Figure 1). Individuals were captured at their nests using a hand net, weighed, and physically restrained for clinical assessment and blood collection. Blood samples were obtained via venipuncture of the metatarsal or ulnar vein of the individuals using 24- or 26-gauge needles and 3 mL syringes. The total volume collected (maximum 1% of the individual's body weight) was divided between EDTA and serum tubes, and anticoagulant-free blood smears were immediately prepared. Each individual underwent a physical examination, which included assessment of hydration status (analysis of eyes position and prominence and mucous membrane moistness), oral mucous membrane coloration, plumage condition and body condition score (11). Following sampling and clinical examination, individuals were fitted with uniquely numbered metal leg bands and released at the capture site. All blood samples were immediately refrigerated and sent to the laboratory for analysis, within a maximum of 6–7 h after collection. Samples were processed at the Veterinary Clinical Pathology Laboratory, Universidade Federal Fluminense.

### 2.3 Hematological analysis

**Figure 2:**
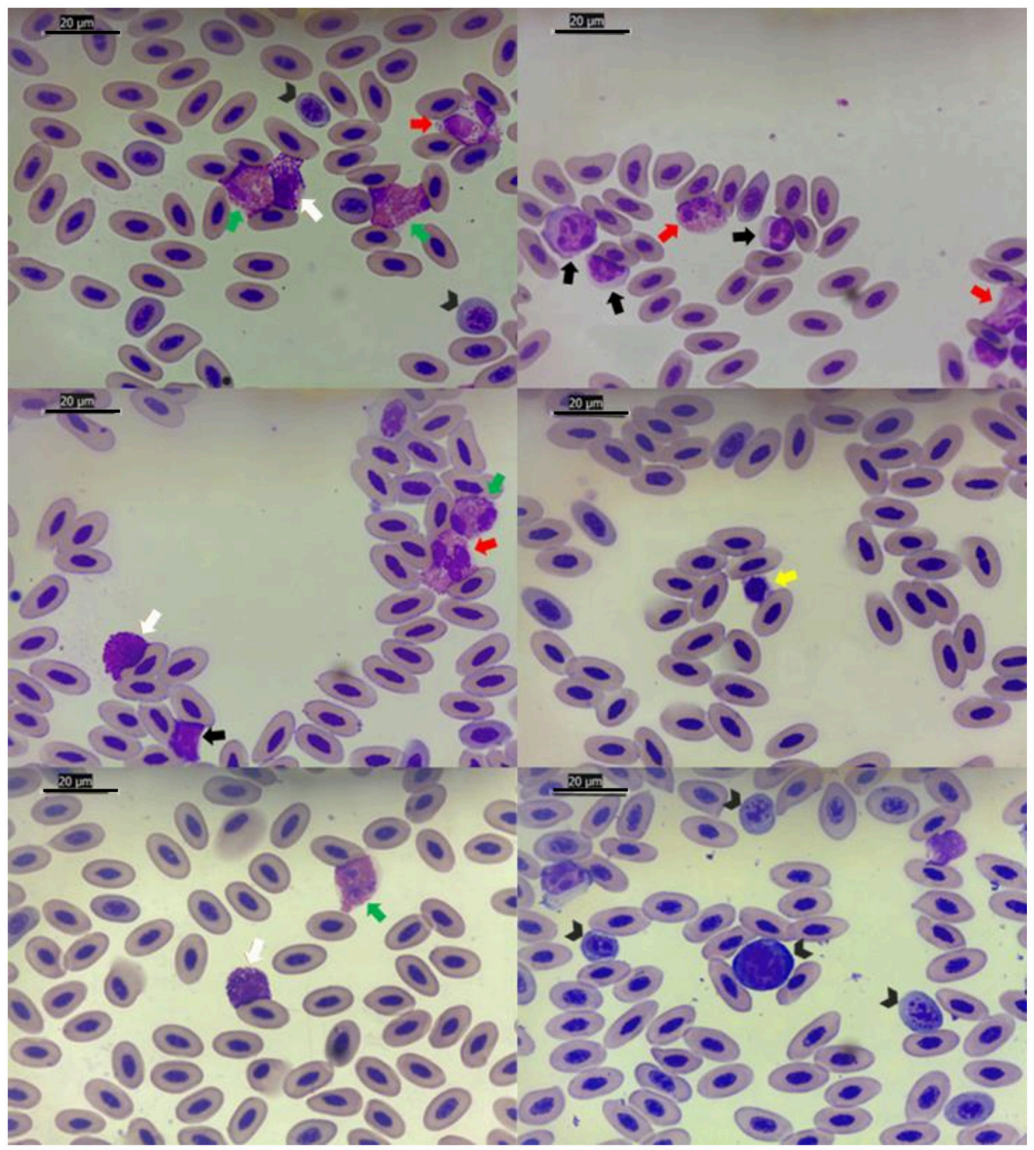
Images highlighting different types of blood cells in brown booby, *Sula leucogaster* (Boddaert, 1783), from Cagarra Island, Rio de Janeiro, Brazil. Rosenfeld^®^ stain, 1,000 × original magnification. Black arrowheads: different levels of immature erythrocytes. Red arrows: eosinophils. Green arrows: heterophils. White arrows: basophils. Black arrows: lymphocytes. Yellow arrows: thrombocytes.

**Figure 3:**
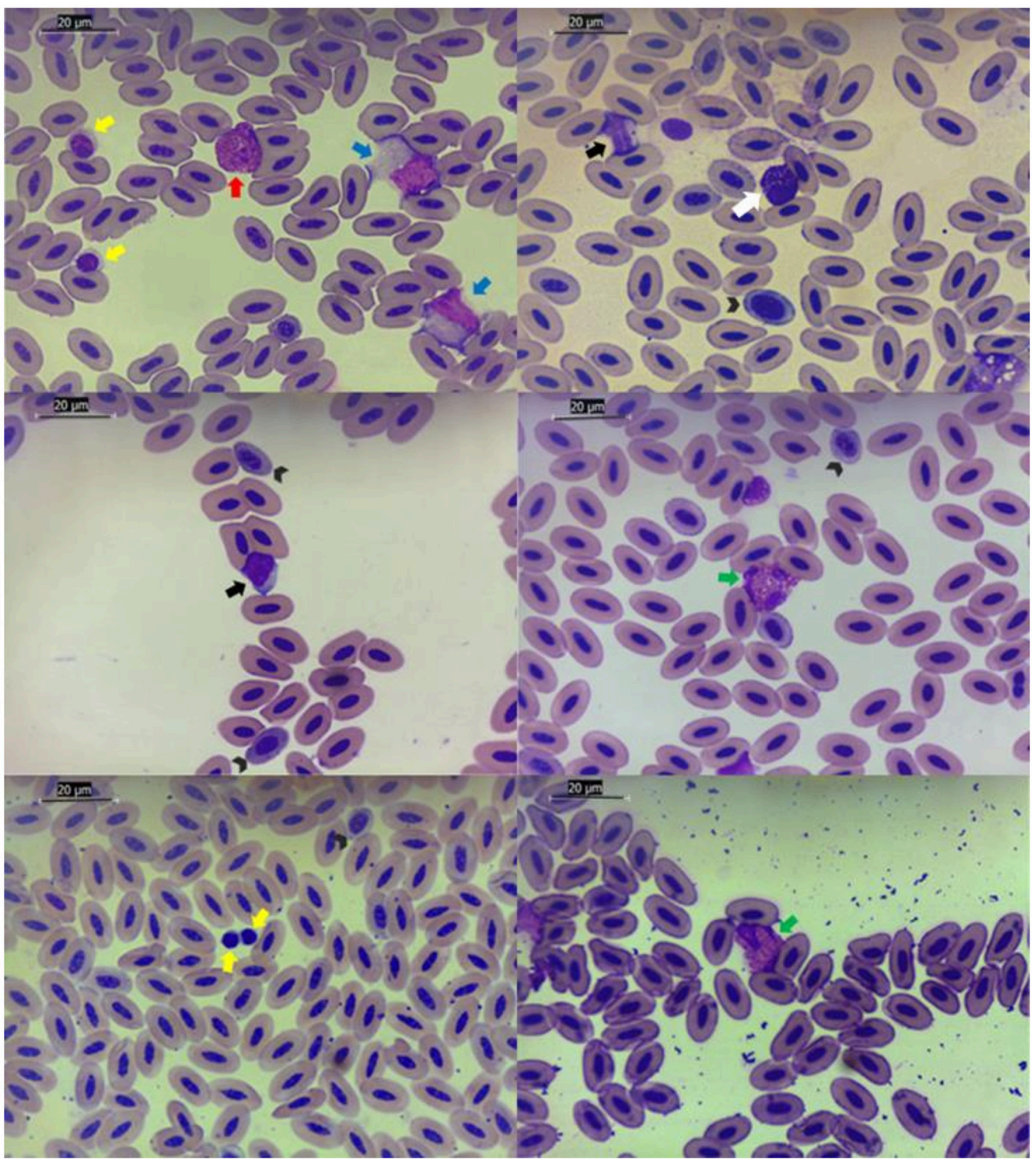
Images highlighting different types of blood cells in magnificent frigatebirds*, Fregata magnificens* (Linnaeus, 1758), from Cagarra Island, Rio de Janeiro, Brazil. Rosenfeld^®^ stain, 1,000 × original magnification. Black arrowheads: different levels of immature erythrocytes. Red arrows: eosinophils. Green arrows: heterophils. White arrows: basophils. Black arrows: lymphocytes. Yellow arrows: thrombocytes. Blue arrows: monocytes.

Packed cell volume (PCV or hematocrit), erythrocyte, hemoglobin concentration (Hb), leukocyte, leukocyte differential count, thrombocyte and total plasma protein (TPP) were determined. Blood cell counts were determined manually using a hemocytometer (Neubauer chamber) with a Natt and Herrick stain (diluted 1:100). The PCV was obtained with the microhematocrit method after centrifugation for 5 min at 14,490 g (Microhematocrit centrifuge, SPIN 1000, Microspin, Jaboticabal, SP, Brazil). Hemoglobin concentration was measured using a colorimetric method (Hemoglobin, Labtest Diagnóstica S. A., Minas Gerais, Brazil) by the cyanmethemoglobin method in a semi-automated analyzer (Bio 2000, Bioplus, São Paulo, Brazil). The total plasma protein concentration was estimated by refractometry (Manual refractometer RHC-200, Megabrix, Paraná, Brazil). The leukocyte differential count and cell morphology were assessed by evaluating 100 leukocytes on Rosenfeld^®^-stained blood smears. The images provided in the study to support hematological characterization were obtained via Leica Application Suite Version 3.4.0© from Leica Microsystems (Switzerland) Limited (Figures 2, 3).

### 2.4 Biochemical analysis

Biochemical analytes were measured using an automatic biochemical analyzer (CM250 – Wiener^®^ Lab, São Paulo, Brazil) with serum obtained from serum tubes after centrifugation (Centrifuge 80-2B, Centrilab^®^, São Paulo, Brazil) at 2,325 g for 10 min. Analyzer was previously calibrated (Calibra - Labtest^®^) and checked with control serum containing known normal and pathological values (Qualitrol 1 and 2, Labtest^®^). Globulin (Glo) was calculated using a standard formula (Globulin = Total Plasma Protein - Albumin). Uric acid (UA), albumin (Alb), aspartate aminotransferase (AST), alanine aminotransferase (ALT), total proteins, and creatine kinase (CK) were measured using commercial kits (Labtest^®^).

### 2.5 Exclusion criteria

#### 2.5.1 Sample-related exclusion criteria

**Figure 4:**
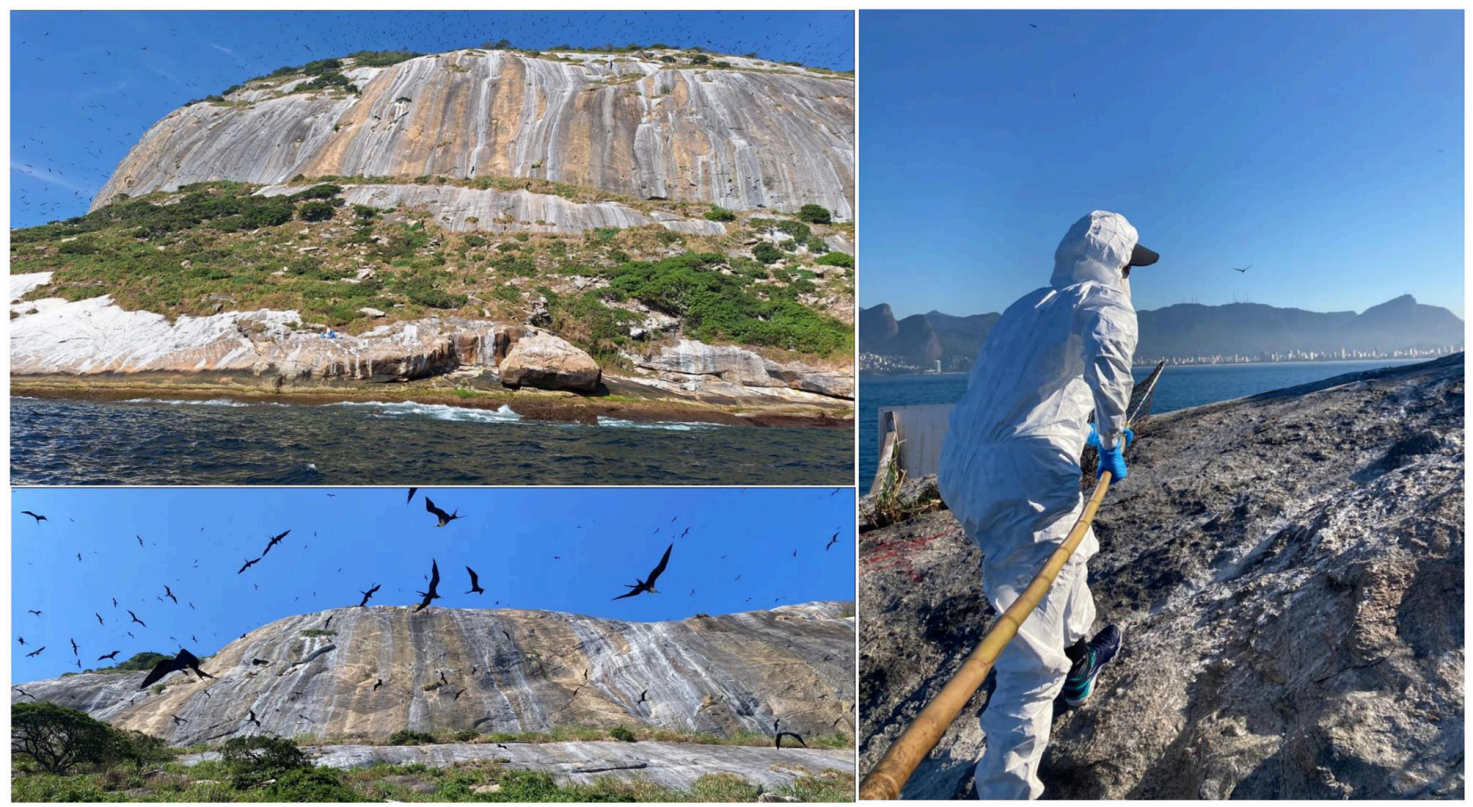
Photographs taken during field activities in the Cagarras Archipelago, Rio de Janeiro, Brazil, illustrating capture and sampling locations and efforts involving brown boobies, *Sula leucogaster* (Boddaert, 1783), and magnificent frigatebirds, *Fregata magnificens* (Linnaeus, 1758) for the establishment of hematological and biochemical reference intervals. Photographs by Sergio Pires Jordão and Joana Medina Maçaira.

Because access to the islands was highly restricted (Figure 4)—requiring approximately 1 h of boat travel, subsequent swimming and rock climbing, and an additional 1-h drive from the landing site to the laboratory—a substantial proportion of samples were considered unsuitable for the establishment of hematological and biochemical RIs. Therefore, the following exclusion criteria related to sample quality and processing were applied: (i) compromised sample quality, including clotting (15%; 12/80), hemolysis (12.5%; 10/80), or lipemia (2.5%; 2/80); and (ii) insufficient sample volume to perform all proposed analyses (8.75%; 7/80).

#### 2.5.2 Individual-related exclusion criteria

In addition, exclusion criteria related to individual health status were applied based on on-site clinical evaluation: (iii) clinical abnormalities detected during physical examination, such as low body condition score or dehydration (7.5%; 6/80); and (iv) presence of hemoparasites observed in blood smears (3.75%; 3/80).

After applying all exclusion criteria, of the 80 individuals initially sampled (42 brown boobies and 38 magnificent frigatebirds), 40 (20 per species) individuals were included in the establishment of the proposed hematological RIs and, 52 (28 brown boobies and 24 magnificent frigatebirds) individuals were included in the biochemical RIs analyses.

### 2.6 Statistics

The hematological and biochemical RIs established in the present study followed the guidelines of the American Society for Veterinary Clinical Pathology (ASVCP) (12). Although current guidelines recommend a minimum of 120 individuals for the establishment of RIs, they also acknowledge that achieving such sample sizes is often challenging in veterinary studies involving wild-caught species, as in the present study. Accordingly, data normality was assessed, and parametric methods were applied for RIs estimation, following the ASVCP recommendations for sample sizes ranging from ≥20 to <40 individuals. In accordance with ASVCP recommendations, extreme values were examined during the assessment of data distribution but were not excluded unless there was clear evidence of analytical error and/or biological inconsistency. As previously described in recent studies (13), the normality of each parameter was assessed using both Kolmogorov-Smirnov (KS) and Shapiro-Wilk (SW) tests (14). Non-Gaussian variables were subjected to Box-Cox transformation, after which the KS and SW tests were reapplied (results for both tests were identical before and after transformation). All parameters were then normalized, and RIs were calculated using the parametric Student's *t*-test-based method. To compare hematological and biochemical parameters between the two species, one-way analysis of variance (ANOVA) was performed, followed by Tukey's *post-hoc* test. A *p-value* <0.05 was considered statistically significant.

## 3 Results

The hematological RIs for MoNa Cagarras' population of brown boobies is based on blood analysis of 20 individuals, and the biochemical RIs reflect data from 28 birds (Tables 1, 2, respectively).

**Table 1:** Hematologic reference intervals (*n* = 20), according to Gaussian distribution, for brown booby, *Sula leucogaster* (Boddaert, 1783) population of Cagarras Archipelago, Rio de Janeiro – RJ, Brazil.

Parameter^*^	**Mean**	**SD**	**Min**	**Max**	**RI**	**L**	**U**
PCV (%)	45	3.2	38	49	38.5–51.4	36.4–41.3	49.8–52.4
Erythrocytes (10^6^/μL)	2.2	0.5	1.3	3.1	1.3–3.2	1–1.7	2.9–3.4
Leukocytes (/μL)	13,474.1	8,268.4	2,300	36,800	0–30,010.8	0–2,762.5	20,285.7–37,320.3
Basophils (/μL)	613.9	374.7	0	1,286.7	0–1,363.4	0–74.1	1,114.8–1,535.9
Eosinophils (/μL)	1,291.5	993.4	88.4	3,312	0–3278.4	0.0	2,419.7–3,901.6
Heterophils (/μL)	7,844.7	3,866.5	1,380	14,850	111.7–15,577.8	0–2,349.1	12,895.7–17,603.2
Lymphocytes (/μL)	1,417.6	1,139.8	159.6	4,416	0–3,697.1	0.0	2,497–4,619.1
Monocytes (/μL)	2,305.2	3,389.4	176.8	13,248	0–9,083.9	0.0	4,109.1–12,734.1
Thrombocytes (/μL)	49,535.9	23,131.9	8,850	123,200	3,272–95,799.8	0–23,577.8	71,297.2–119,506.8

^*^Hb, hemoglobin; PCV, packed cell volume; SD, standard deviation; Min, minimum; Max, maximum; RI, reference interval; L, 90% confidence interval for lower limit; U, 90% confidence interval for upper limit.

**Table 2:** Biochemical reference intervals (*n* = 28), according to Gaussian distribution, for brown booby, *Sula leucogaster* (Boddaert, 1783) population of Cagarras Archipelago, Rio de Janeiro – RJ, Brazil.

Parameter^*^	**Mean**	**SD**	**Min**	**Max**	**RI**	**L**	**U**
ALT (UI/L)	46.1	17	21	92	12.2–80	6–21.4	66–91.1
AST (UI/L)	256.8	63.6	144	393	129.6–384	107–158.7	345.7–414.6
CK (UI/L)	1,350.1	717.3	678	3,644	0–2,784.6	0–374.4	1,944.6–3,364.6
Total protein (g/dL)	4.4	2	2.9	13.8	0.4–8.4	0–2.8	5–11.4
Alb (g/dL)	1.5	0.6	0.9	4.1	0.3–2.6	0–1	1.7–3.4
Glo (g/dL)	2.9	1.5	2	9.7	0–5.9	0–1.7	3.5–8
Alb/Glo	0.5	0.1	0.4	0.9	0.3–0.8	0.3–0.4	0.7–0.8
Uric acid (g/dL)	19.8	9.7	3.1	45.4	0.3–39.3	0–5.2	33.2–44.3

^*^ALT, alanine aminotransferase; AST, aspartate aminotransferase; Alb, albumin; Glo, globulin; CK, creatine kinase; SD, standard deviation; Min, minimum; Max, maximum; RI, reference interval; L, 90% confidence interval for lower limit; U, 90% confidence interval for upper limit.

The hematological RIs for MoNa Cagarras' population of magnificent frigatebirds is based on blood analysis of 20 individuals, and the biochemical RIs reflect data from 24 birds (Tables 3, 4, respectively).

**Table 3:** Hematologic reference intervals (*n* = 20), according to Gaussian distribution, for magnificent frigatebirds, *Fregata magnificens* (Linnaeus, 1758) population of Cagarras Archipelago, Rio de Janeiro – RJ, Brazil.

Parameter^*^	**Mean**	**SD**	**Min**	**Max**	**RI**	**L**	**U**
PCV (%)	47.8	5	35	55	37.7–57.8	33.7–43.8	54.3–59.7
Erythrocytes (10^6^/μL)	2.5	0.6	1.6	3.6	1.3–3.6	1.1–1.6	3.1–4
Hb concentration (g/dL)	12	1.7	8.5	14.4	8.6–15.4	7.7–9.7	14.6–16
Leukocytes (/μL)	17,898.5	10,060.6	6,400	45,820	0–38,019.8	0–3,537.3	27,826–46,628.8
Basophils (/μL)	262.3	336.1	0	1,216.8	0–934.6	0.0	537–1,219.2
Eosinophils (/μL)	2,749.7	2,741.1	326.8	10,538.6	0–8,231.9	0.0	4,717.4–10,691.9
Heterophils (/μL)	7,306.7	5,372.2	1,744.2	25,490.4	0–18,051	0–992.7	10,875.3–24,489.5
Lymphocytes (/μL)	7,114.9	3,714.1	2,131.8	17,411.6	0–14,543.1	0–1,983.5	10,987.4–17,587
Monocytes (/μL)	513.8	495.8	78	2,291	0–1,505.3	0.0	813.5–2,137.2
Thrombocytes (/μL)	42,251.5	9,579.1	26,880	60,750	23,093.2–61,409.8	19,517.1–28,027.2	54,394.6–66,599.7

^*^Hb, hemoglobin; PCV, packed cell volume; SD, standard deviation; Min, minimum; Max, maximum; RI, reference interval; L, 90% confidence interval for lower limit; U, 90% confidence interval for upper limit.

**Table 4:** Biochemical reference intervals (*n* = 24), according to Gaussian distribution, for magnificent frigatebirds, *Fregata magnificens* (Linnaeus, 1758) population of Cagarras Archipelago, Rio de Janeiro – RJ, Brazil.

Parameter^*^	**Mean**	**SD**	**Min**	**Max**	**RI**	**L**	**U**
ALT (UI/L)	27.7	8.2	14	45	11.3–44.1	8.5–15.6	38.4–48.9
AST (UI/L)	316.8	87.1	184	523	142.5–491	106–193.9	421.2–547.6
CK (UI/L)	1,801.5	993.7	654	4,641	0–3,788.8	0–295.9	2,842.9–4,578.8
Total protein (g/dL)	3.5	0.5	2.4	4.4	2.5–4.6	2.2–2.8	4.3–4.8
Alb (g/dL)	0.9	0.3	0.4	1.8	0.4–1.4	0.3–0.6	1.2–1.7
Glo (g/dL)	2.6	0.5	1.7	3.4	1.7–3.5	1.5–2	3.3–3.7
Alb/Glo	0.4	0.1	0.2	0.8	0.1–0.6	0–0.2	0.5–0.7
Uric acid (g/dL)	12.1	8.5	2	31.1	0–29	0.0	21.2–34.8

^*^ALT, alanine aminotransferase; AST, aspartate aminotransferase; Alb, albumin; Glo, globulin; CK, creatine kinase; SD, standard deviation; Min, minimum; Max, maximum; RI, reference interval; L, 90% confidence interval for lower limit; U, 90% confidence interval for upper limit.

Comparing hematological parameters of both species, basophils (*p* = 0.00), eosinophils (*p* = 0.03), lymphocytes (*p* = 0.00), monocytes (*p* = 0.02), and PCV (*p* = 0.04) differed significantly among them (Table 5). As shown in Table 6, among the biochemical parameters, only CK (*p* = 0.06) and globulin (*p* = 0.34) did not differ significantly among species.

**Table 5:** One-way ANOVA and Tukey's *post-hoc* test comparing hematologic parameters among brown booby, *Sula leucogaster* (Boddaert, 1783) (*n* = 20) and magnificent frigatebird, *Fregata magnificens* (Linnaeus, 1758) (*n* = 20) populations from Cagarras Archipelago, Rio de Janeiro – RJ, Brazil.

Parameter^*^	**df**	**MS**	**F**	***p-value* (One-way ANOVA)**	***p-value* (Tukey's *post-hoc* test)**
PCV (%)	1	78.40	4.42	0.04	0.04
Erythrocytes (10^6^/μL)	1	0.47	1.70	0.20	0.20
Hb concentration (g/dL)	1	0.14	0.06	0.81	0.81
Leukocytes (/μL)	1	195,753,153.60	2.31	0.14	0.14
Basophils (/μL)	1	1,235,993.55	9.75	0.00	0.00
Eosinophils (/μL)	1	21,263,034.94	5.00	0.03	0.03
Heterophils (/μL)	1	2,894,644.44	0.13	0.72	0.72
Lymphocytes (/μL)	1	324,591,475.28	43.01	0.00	0.00
Monocytes (/μL)	1	32,089,599.01	5.47	0.02	0.02
Thrombocytes (/μL)	1	530,624,833.60	1.69	0.20	0.20

^*^Hb, hemoglobin; PCV, packed cell volume; df, degrees of freedom; MS, mean square; F, F statistic (ratio of variance between and within groups).

**Table 6:** One-way ANOVA and Tukey's *post-hoc* test comparing biochemistry parameters among brown booby, *Sula leucogaster* (Boddaert, 1783) (*n* = 28) and magnificent frigatebird, *Fregata magnificens* (Linnaeus, 1758) (*n* = 24) populations from Cagarras Archipelago, Rio de Janeiro – RJ, Brazil.

Parameter^*^	**df**	**MS**	**F**	***p-value* (One-way ANOVA)**	***p-value* (Tukey's *post-hoc* test)**
ALT (UI/L)	1	4,357.70	23.42	0.00	0.00
AST (UI/L)	1	46,467.71	8.19	0.01	0.01
CK (UI/L)	1	2,633,634.29	3.60	0.06	0.06
Total protein (g/dL)	1	9.35	4.09	0.05	0.05
Alb (g/dL)	1	3.90	18.85	0.00	0.00
Glo (g/dL)	1	1.17	0.93	0.34	0.34
Alb/Glo	1	0.34	25.53	0.00	0.00
Uric acid (g/dL)	1	772.02	9.18	0.00	0.00

^*^ALT, alanine aminotransferase; AST, aspartate aminotransferase; Alb, albumin; Glo, globulin; df, degrees of freedom; MS, mean square; F, F statistic (ratio of variance between and within groups).

## 4 Discussion

Establishing reference intervals for hematological and biochemical profiles in wild populations is challenging and often relies on pre-release evaluations of previously hospitalized animals in rehabilitation centers (15). Although these assessments are typically conducted on individuals deemed clinically recovered, reliable RIs should be established using data from free-ranging, apparently healthy populations to enable the early detection of subclinical processes and to guide therapeutic decisions, particularly in species frequently found stranded along the coast (1). Therefore, determining hematological and biochemical RIs for brown boobies and magnificent frigatebirds in their natural habitat is essential.

### 4.1 Hematology of *S. leucogaster* and *F. magnificens*

Previous studies have investigated the hematology and clinical chemistry of *S. leucogaster* (15–17) and *F. magnificens* (7, 16, 18, 19). However, they differed from the present study either in the geographical location or in the *ex-situ* condition of the sampled animals. This study analyzed apparently clinically healthy, *in situ* populations from a reproductive colony of brown boobies and magnificent frigatebirds on the coast of the Rio de Janeiro city, Brazil.

Overall, our hematological results are comparable to those reported in earlier studies. The most notable differences are observed in the total thrombocyte counts, which were higher in our study for both species. This increase may be associated with stress related to capture and sample collection—an influencing factor most likely absent in rehabilitated birds that are, to some extent, habituated to human interaction. Another major difference was found in total leukocytes, also higher in our population compared to hospitalized individuals, but similar to those reported for free-ranging brown boobies, and particularly to the study conducted on *F. magnificens* along the Rio de Janeiro coast, although in rehabilitation centers. This variation could be related to the use of medications or ongoing recovery processes in birds from rehabilitation centers.

### 4.2 Clinical chemistry of *S. leucogaster* and *F. magnificens*

Among the biochemistry analytes, our results presented higher values for UA and CK, for both species, and ALT for frigatebirds, when compared to those from rehabilitated individuals, but similar to those described in free-ranging individuals. This increase may be related to dietary and physiological differences. In their natural habitat, brown boobies and magnificent frigatebirds (both piscivorous) typically consume natural, high-protein prey and experience irregular feeding intervals, both of which can transiently increase UA, CK, and ALT concentrations (20, 21). Additionally, capture-related stress and recent feeding events may further contribute to these elevations (17). Conversely, for *S. leucogaster*, ALT, AST, and albumin levels were lower in the present study, which may reflect differences between individuals previously exposed to pathological or metabolic alterations, even if clinically recovered, and truly healthy *in situ* animals (22). Other parameters for *F. magnificens* did not vary significantly from previous studies.

### 4.3 Final considerations

In the present study, we acknowledge that the proposed RIs could be further strengthened with broader datasets, as they may be influenced by factors such as age, sex, feeding, and reproductive status (23). Due to the limited number of individuals evaluated, these variables could not be statistically assessed. Nevertheless, our findings provide an important foundation for future research on these species.

Conducting health assessments in wildlife populations remains inherently challenging and complex, particularly when working with free-ranging individuals in their natural habitat. Despite these limitations, the present study contributes with novel and regionally relevant data. The hematologic and biochemical reference intervals established here for free-ranging populations of *S. leucogaster* and *F. magnificens* along the coast of Rio de Janeiro, Brazil, in their natural habitat, represent a pioneering effort. These data provide valuable information for clinicians and researchers, supporting both clinical evaluation and the management of animals frequently admitted to rehabilitation and conservation programs.

## Data Availability

The original contributions presented in the study are included in the article/supplementary material, further inquiries can be directed to the corresponding authors.
